# Comprehensive Rehabilitation of a Patient With Right Hemiplegia: A Case Report

**DOI:** 10.7759/cureus.52065

**Published:** 2024-01-10

**Authors:** Maithili S Deshmukh, Pallavi Harjpal, Vaishnavi M Thakre, Aditi Dandekar, Sanjivani S Bangde

**Affiliations:** 1 Neurophysiotherapy, Ravi Nair Physiotherapy College, Datta Meghe Institute of Higher Education and Research, Wardha, IND

**Keywords:** rehabilitation, physiotherapy, hemiplegia, stroke, middle cerebral artery

## Abstract

Stroke is a common cause of death and neurological impairment. The largest artery in the brain is the middle cerebral artery. When this artery suffers a stroke from an abrupt interruption or cessation of blood flow, tissue dies, and severe, potentially irreversible brain damage occurs. Comprehensive assessments of stroke patients are required for proper care management, evaluation of interventions, and assessment of outcomes. With the development of new, efficient stroke treatments, it is of the utmost significance for nurses to take advantage of the chance to document stroke impairments and disabilities to track recovery and make plans for re-entry into society.

We describe the case of a 50-year-old man with right hemiplegia. The brain CT made visible the left front parietotemporal region and the left insular cortex of the acute left-sided middle cerebral artery infarct. The patient received immediate medical attention. After the early stabilization of acute symptoms, physiotherapy treatment was started. The physiotherapy intervention given in this case enabled the patient to have a speedy and effective recovery. It helped improve his motor impairments and quality of life. Post-rehabilitation, the patient became independent in daily activities like brushing, bathing, eating, etc.

## Introduction

During a stroke, insufficient blood flow to the brain leads to cell death, causing a high risk of mortality or disability. The lateral sulcus, connecting the frontal and temporal lobes and part of the circle of Willis, often experiences pathology affecting the brain's most commonly affected blood vessel [1]. A stroke in the middle cerebral artery can cause problems with the senses and vision. If it affects the right side of the artery, the symptoms show up on the left side of the body [2]. Having high cholesterol, smoking, and being around tobacco smoke can make one more likely to have a stroke. High blood pressure, obesity, diabetes, and not being physically active also increase the risk by causing damage to blood vessels and promoting atherosclerosis [3].

Hemiplegia is when one side of the body can't move or feel after a stroke. It can cause issues like apraxia, neglect of one side, lack of awareness about the condition, decreased sensation, and problems with muscle tone and reflexes. When the upper limbs and face lose sensation and there's spastic hemiplegia on the opposite side of the affected area, it's often due to a stroke blocking the middle cerebral artery [4]. Aphasia can happen when the language center in the dominant hemisphere of the brain is affected [5]. Brain MRI and CT scans are frequently performed to help with detection [6]. These impairments hinder an individual from carrying out daily tasks.

Proprioceptive neuromuscular facilitation (PNF) is based on stimulating the proprioceptors to get the most out of the neuromuscular system. This technique has been used to treat several neurological and musculoskeletal disorders. After an acute stroke, the D2 flexion pattern of PNF can be used [7]. Physical therapy is generally very effective after a stroke [8]. Stroke complications can include hemiplegia and hemiparesis, which mean losing the ability to move certain muscles, usually on one side of the body. Other challenges may include trouble swallowing and speaking, memory loss, and problems with thinking, decision-making, reasoning, and understanding. Emotional issues like depression can also develop in stroke survivors.

One of the essential fields in stroke rehabilitation is physiotherapy [9]. Strong evidence supports physical therapy interventions focusing on intense, repetitive, task-specific training during all post-stroke phases [10]. In this report, we describe the use of physiotherapeutic interventions such as progressive exercises to increase strength and endurance, Kegel's exercises to increase the strength of pelvic floor muscles, electromechanically assisted gait training for gait rehabilitation that can be used in non-ambulatory patients, and exercises for facial muscle palsy. One of the most common functional tasks is standing up from a seated position, which is also important for independent living and preventing falls [11]. Asymmetric standing balance is a common symptom in stroke patients, which affects stability limits and makes it challenging to regain functional independence. This is addressed with electromechanical-assisted gait training [12].

After a stroke, people typically have low physical fitness levels. It's uncertain whether improving physical fitness post-stroke reduces disability [13]. Ambulation after a stroke may benefit from gait training [14]. Gait-training tools with electromechanical and robotic assistance are used to improve walking post-stroke [15]. Physiotherapy helps rehabilitate patients and improve their quality of life. This case report reveals how physiotherapeutic interventions such as progressive exercises, Kegel's exercises, electromechanical-assisted gait training, and exercises for facial muscle palsy can treat stroke patients and enhance their motor skills.

## Case presentation

We report the case of a 50-year-old male patient, a mechanic, who complained of weakness in his right upper and lower limbs as well as a mouth deviation. He also reported having a gradually progressive ache in his right shoulder, which aggravates with shoulder movement and relieves with rest. The patient had undergone certain investigations, such as MRI and CT, and was diagnosed with a middle cerebral artery stroke. Presently, the patient complains of generalized weakness and pain in the right shoulder. He neither gives a history of any comorbidities nor bladder or bowel complaints. After investigations, physiotherapy rehabilitation was commenced with a tailor-made protocol for the patient.

Clinical findings

On examination, the patient was conscious, cooperative, and well-oriented to place, time, and person. The patient was hemodynamically stable. He was mesomorphic in build. He was aphasic, but his vision and hearing were intact. During the neurological evaluation, sensations were intact. Muscle strength was reduced in the lower and upper limbs. All deep tendon reflexes were diminished on the right side (Table 1).

**Table 1:** Deep tendon reflex 1+: Diminished reflex, 2+: Normal reflex

Reflexes	Right side	Left side
Biceps jerk	2+	2+
Triceps jerk	1+	2+
Supinator	1+	2+
Knee jerk	1+	2+
Ankle jerk	1+	2+

Diagnostic findings

**Figure 1:**
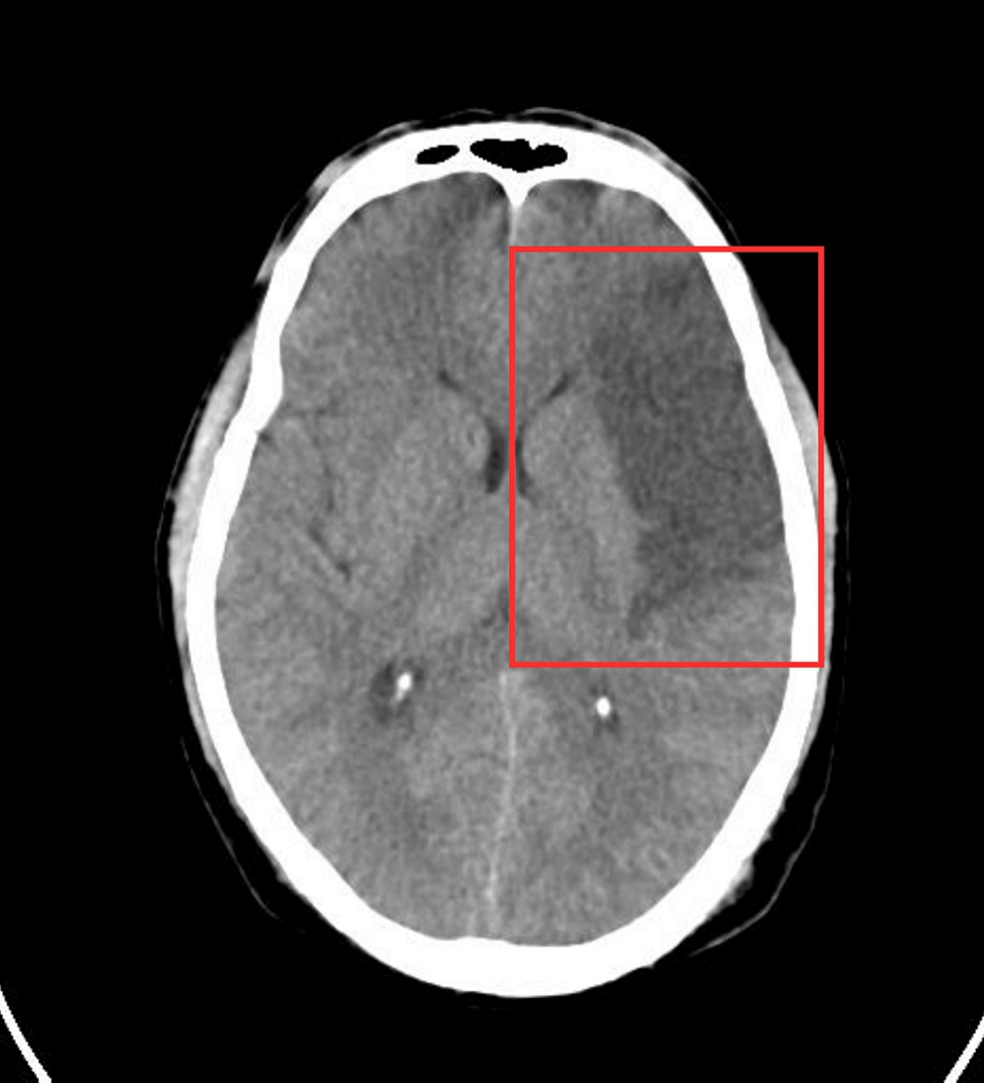
CT brain The rectangle demarcates the acute left-sided MCA infarct in the left frontoparietal-temporal region and left insular cortex. MCA: Middle cerebral artery

A CSF examination along with a blood count (CBC) assessment were done. A CT scan of the head revealed a middle cerebral artery stroke of the frontoparietal-temporal region on the left side (Figure 1).

Physiotherapeutic interventions

**Figure 2:**
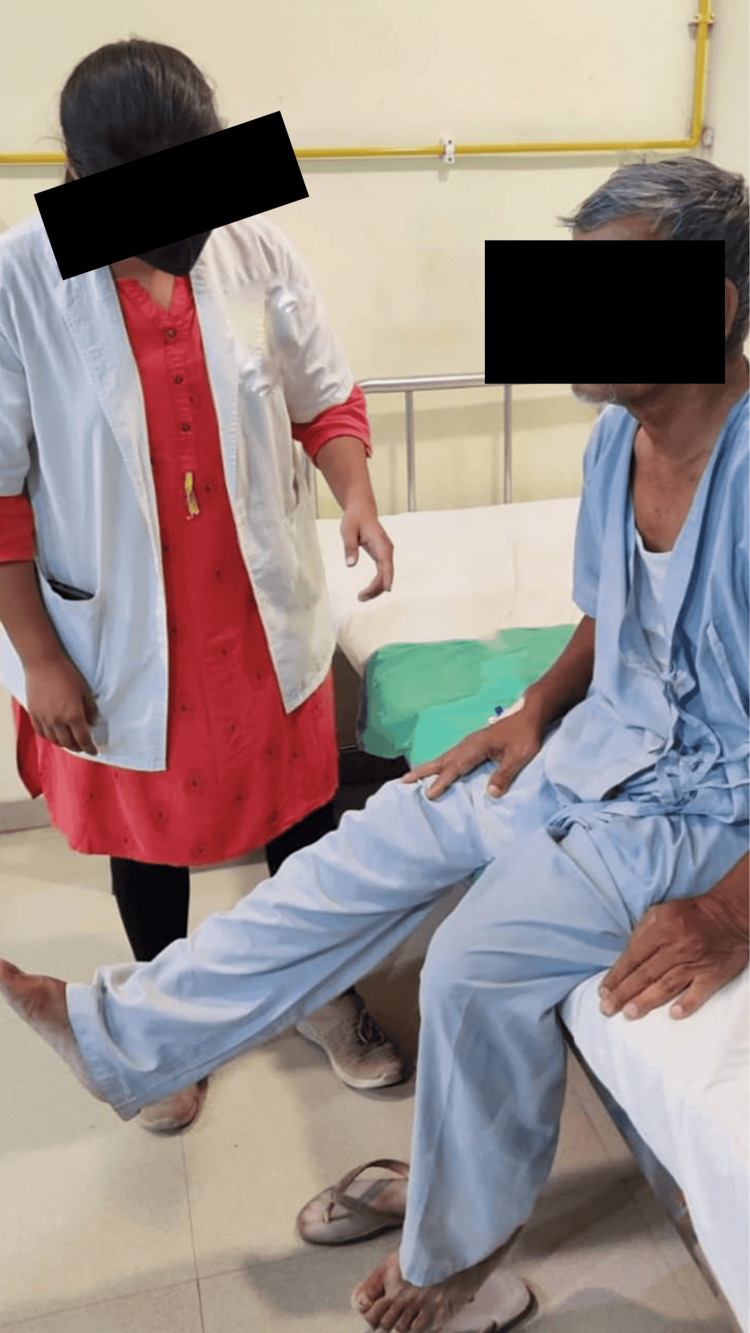
Patient performing dynamic quadriceps exercise

**Figure 3:**
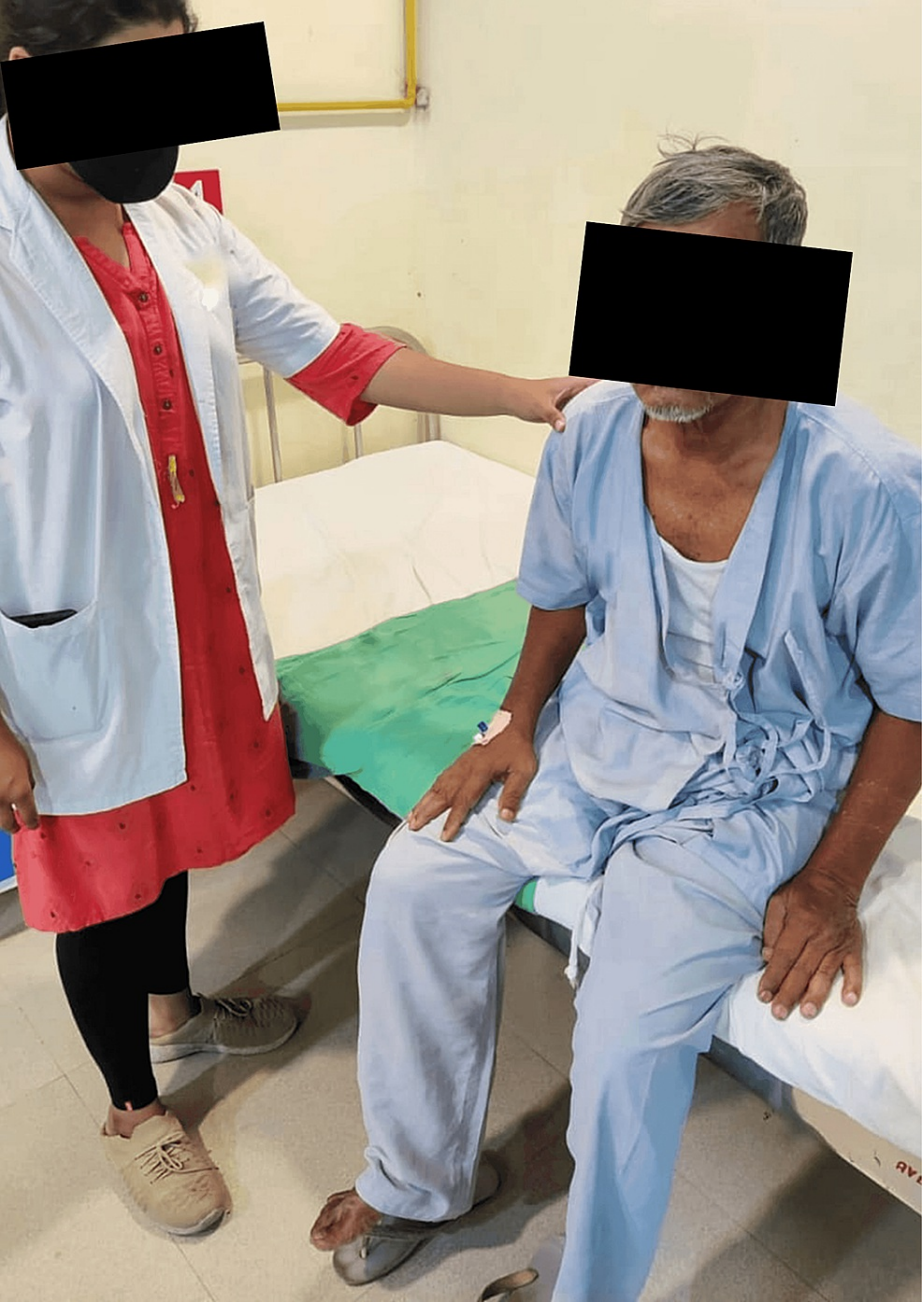
Patient performing bedside sitting

**Figure 4:**
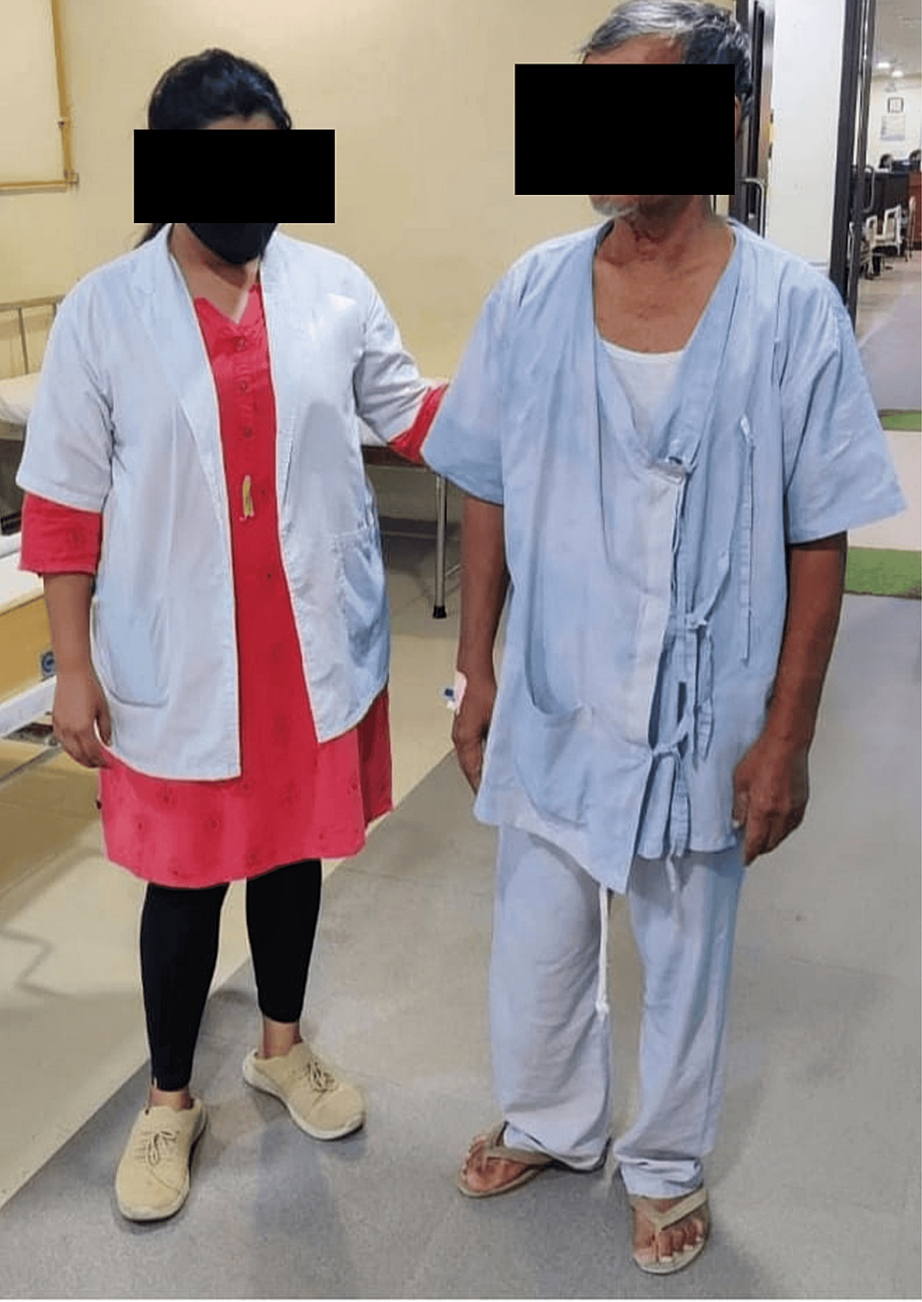
Patient undergoing gait training

The patient was treated with a multidisciplinary approach that was used to improve his recovery. The physiotherapy interventions used for the patient are shown in Table 2 and Figures 2-4.

**Table 2:** Physiotherapeutic intervention PNF: Proprioceptive neuromuscular facilitation, reps: repetitions, Quads: Quadriceps

Complaints	Goals	Physiotherapy	Dosage
Weakness of right side upper and lower limb	To increase strength, endurance	Progressive exercises with the resistance band in a PNF D2 flexion pattern and dynamic quads	10 reps in 1 set
Facial palsy	To make the facial muscles stronger	Exercise program at home for the facial muscles	10 reps in 1 set
Incontinence	To increase pelvic floor muscle strength	Kegel’s exercise	10 reps in 1 set
Inability to change position and posture	To make the patient independent in changing position and posture	Arm placement aids, such as the lap tray, are employed by those who are at risk of shoulder dislocation. Introduction and practice in using the correct manual. The person experiencing the stroke and their family should be taught.	10 reps in 1 set
Unable to maintain balance	To maintain balance	Training for unsupported bedside sitting and maintaining balance. Also, train the individual to maintain balance while standing. A wobble board can be used.	10 reps in 1 set
Gait disturbances	Gait improvement	Electromechanical-assisted gait training for gait rehabilitation can be used in non-ambulatory patients.	10 reps in 1 set

Outcome measures

After four weeks of treatment, the following outcome measures were assessed under the supervision of the therapist. Pre- and post-treatment scores are shown in Tables 3-4.

**Table 3:** Outcome measures

Outcome measures	Pre-treatment score	Post-treatment score
Berg balance test	36/56	45/56
Functional independence major	71/126	96/126
Dynamic gait index	11	20
Modified Rankin scale	3/6	1/6

**Table 4:** Manual muscle testing of upper limb and lower limb 0: No contraction; 1: Flickering contraction; 2: Full ROM with gravity eliminated; 3: Full ROM against gravity; 4: Full ROM against gravity, moderate resistance; 5: Full ROM against gravity, maximal resistance MMT: Manual muscle testing, ROM: Range of motion

MMT (joints)	Pre-treatment score	Post-treatment score
Upper limb		
Shoulder flexors	3/5	4/5
Shoulder extensors	3/5	4/5
Shoulder abductors	3/5	4/5
Shoulder internal rotators	3/5	4/5
Shoulder external rotators	3/5	4/5
Elbow flexors	3/5	4/5
Wrist flexors	3/5	4/5
Wrist extensors	3/5	4/5
Lower limb		
Hip flexors	3/5	4/5
Hip extensors	3/5	4/5
Hip abductors	3/5	4/5
Hip internal rotators	3/5	4/5
Hip external rotators	3/5	4/5
Knee flexors	3/5	4/5
Ankle flexors	3/5	4/5
Ankle extensors	3/5	4/5

## Discussion

A stroke is defined as an abrupt localized neurologic impairment brought on by bleeding [16]. The most prevalent kind of strokes are middle cerebral artery strokes. The entire lateral side of the brain receives blood flow from the middle cerebral artery. If major regions are affected, this stroke may leave the patient with lifelong, severe impairments [17].

Neurological rehabilitation makes use of a wide variety of treatment methods and strategies from various perspectives. The purpose of rehabilitation is to enable patients to regain their functional abilities; each patient will have different rehabilitation goals. Most studies have found that real-world motor training and routine motor practice are beneficial for stroke patients' motor recovery [18]. In hemiplegia, recovery typically happens from proximal to distal sequence, with finger or shoulder movements recovering last or not at all [19]. The number of randomized controlled trials, including physical rehabilitation for stroke, has increased significantly over the past 10 years. The national clinical guidelines recommend 45 minutes at least of physical therapy every day, as long as rehabilitation goals are in place and the patient is capable of handling this degree of effort.

For stroke patients, some physical interventions include early mobilization, treadmill training, positioning, mobility, gait training, balance, hydrotherapy, strength training, mirror therapy, cardiorespiratory training, electro-mechanical gait-assisted training, etc. One of the main fields in the multidisciplinary treatment of stroke is physical therapy. In all stages after a stroke, there is compelling evidence to support physical therapy interventions that prioritize severe, repeated tasks and task-specific training. The majority of the impacts are limited to the functions and activities that have been trained. Middle cerebral artery stroke rehabilitation can be time-consuming and intensive after a stroke. Physical, occupational, and speech therapy may be used.

Physiotherapeutic interventions used in this case are progressive exercises with resistance, patterns of proprioceptive neuromuscular facilitation (D2 flexion), and dynamic quads to increase the strength of muscles; facial exercises for strengthening of facial muscles; gait rehabilitation for gait improvement; and sit-stand exercises for maintaining balance. Improving balance control, motor function, and the patient's ability to work safely in an active environment are the primary goals of stroke recovery [20].

## Conclusions

The physiotherapy interventions introduced to our patient were quite successful; he recovered quickly and well with an improvement in quality of life. Physiotherapy treatment enhances the strength and re-educates the motor functions in the individual. The rehabilitation program improves the patient's health and motor skills. And the patient would be independent in doing activities of daily life. Better results depend on an early diagnosis and the timely initiation of appropriate therapy. Further understanding of this case's middle cerebral artery stroke is crucial. The neurological examination was centered on any impairments of vision, issues with language, motor weakness, or sensory loss. Additionally, the decision to undergo surgery was made to improve long-term management in addition to providing instant relief while collaborating with immunosuppressive medication. The patient's overall quality of life was taken into consideration, with a primary focus on enhancing results and relieving the severe symptoms related to his diagnosis.
